# A Review of Epidemiology and Cancer Biology of Malignant Melanoma

**DOI:** 10.7759/cureus.15087

**Published:** 2021-05-18

**Authors:** Matthew G Davey, Nicola Miller, Niall M McInerney

**Affiliations:** 1 Surgery, National University of Ireland Galway, Galway, IRL; 2 Plastic, Aesthetic, and Reconstructive Surgery, Galway University Hospitals, Galway, IRL

**Keywords:** melanoma, skin cancer, oncology, biology, epidemiology

## Abstract

Malignant melanoma is a neoplasm originating in the melanocytes in the skin. Although malignant melanoma is the third most common cutaneous cancer, it is recognized as the main cause of skin cancer-related mortality, and its incidence is rising. The natural history of malignant melanoma involves an inconsistent and insidious skin cancer with great metastatic potential. Increased ultra-violet (UV) skin exposure is undoubtedly the greatest risk factor for developing cutaneous melanoma; however, a plethora of risk factors are now recognized as causative. Moreover, modern oncology now considers melanoma proliferation a complex, multifactorial process with a combination of genetic, epigenetic, and environmental factors all known to be contributory to tumorgenesis. Herein, we wish to outline the epidemiological, molecular, and biological processes responsible for driving malignant melanoma proliferation.

## Introduction and background

Cutaneous melanoma is a malignancy neoplasm originating in the melanocytes. Melanocytes are found in the basal layer of the epidermis and are responsible for producing a dark pigment known as melanin, which is responsible for giving skin its color [1,2]. Melanoma is the third most common cutaneous malignancy behind basal cell and squamous cell carcinomas, making up less than 5% of cases [3,4]. In spite of this, it remains the most aggressive skin carcinoma and is associated with the vast majority of skin cancer-related mortality with an overall mortality rate in excess of 10% [5,6]. The natural history of cutaneous melanoma depicts an aggressive disease, which increased metastatic potential based upon primary cancer Breslow thickness, degree of tumor ulceration, and metastasis to local lymphatic nodal basins [7]. The primary aim of this review article was to outline the epidemiological and biological properties underpinning malignant melanoma proliferation. Augmentation of the clinicians’ understanding of the risk factors and molecular properties responsible for tumor development is critical in the attempt to quell our current battle with cancer. Thus, the objective of the current review is to outline the myriad of epidemiological factors contributing to melanoma proliferation and detail the molecular and biological processes driving melanoma oncogenesis.

## Review

Epidemiology and risk factors

Malignant melanoma is the most aggressive skin cancer, and its incidence is rising [8-11]. Malignant melanoma is the 19th most common cancer diagnosis worldwide [12] and is the leading cause of death due to cutaneous malignancy. In spite of only accounting for less than 5% of skin cancer diagnosis, melanoma is responsible for 65% of skin cancer-related deaths [9]. Estimates from the United States quote the average lifetime risk of cutaneous melanoma reaching one in 56 for males and one in 37 for females, while other estimates for the western world report that one in every 50 people are at risk of developing cutaneous melanoma [13,14]. Melanoma can occur in a number of anatomical locations, such as the leptomeninges or uvea; however, cutaneous melanoma is recognized to be accountable for an excess of 90% of diagnoses [15] and a considerable variation in incidence worldwide between continents and countries, with Australian patients having highest rates of diagnosis versus the lowest rates in Central Asia [16]. Highest incidence of cutaneous melanoma occurs in the fair-skinned racial phenotype, combined with geographic areas with intense sun exposure; in the United States, over 98% of cases have been reported in white-skinned people [16]. European populations have evidently less incidence of cutaneous melanoma than the United States and Australia; however, there are discrepancies between countries with regard to melanoma incidence [17]. Moreover, geographical location favors habitants of southern Europe, with countries such as Greece and Cyprus having incidence rates of almost 10% less than that of Switzerland, Denmark, and Norway. Forsea et al. describe an increasing incidence of cutaneous melanoma as latitude increases across mainland Europe. Lower incidence of melanoma is seen in many eastern European countries versus their western counterparts, although acknowledgment must be made for the discrepancies in the consistency in reporting from different cancer registries in different European states. The incidence of melanoma is highest in equatorial regions and decreases as one progresses further north or south of the earth’s equatorial midline, seemingly correlative with the hours of exposure to sunlight in said regions versus areas of higher or lower longitude [18,19].

The incidence of cutaneous melanoma is growing worldwide, and this increase is occurring at a rate faster than other malignancies [20]. The most alarming element of this statistic is the young demographic of patients who are affected by this malignancy. In contrary to other neoplasms such as colon (68 years), lungs (70 years), and prostate (71 years), the median age at diagnosis for cutaneous melanoma patients is just 57 years [21-24]. There are a number of socioeconomic elements to be considered with regard to this younger population undergoing melanoma oncogenesis: one study estimating the lifetime cost of treating melanoma to be in excess of $44,000 (Australian dollars) per cancer, while another illustrating melanoma diagnosis is associated with significant lifestyle changes and difficulty coping with diagnosis [25,26]. With regard to gender, females aged 20-24 years are more likely to be diagnosed with malignant melanoma than males (4:10); however, as age increases males become much more likely to develop melanoma (17:10, aged greater than 65 years). This comes secondary to a sharp increase in the incidence of males approximately at the midpoint of the sixth decade of life [27]. Men diagnosed with cutaneous melanoma have poorer clinical outcomes than their female counterparts, with higher rates of disease recurrence, disease progression, and mortality [28]. Extrapolated rationale for these worse outcomes is the possibility of male patients who are less likely to self-examine for suspicious lesions, more likely to delay presentation, and ultimately present at more advanced stages of disease than their female counterparts [29].

A plethora of risk factors have been identified with melanoma oncogenesis, most of which can be clearly categorized into either environmental and hereditary factors [30,31]. Although we acknowledge in reality that a complex web of these is responsible for malignant melanoma development, increased exposure to ultra-violet (UV) light is undoubtedly recognized as the greatest risk factor for cutaneous melanoma proliferation [32,33]. Modern social culture has seen increasing numbers of people sunbathing for cosmetic purposes, and consequentially an increasing trend in melanoma diagnosis coincides directly with this [34]. As people expose themselves to surplus UV light, the skin’s cellular biology undergoes dysplastic change [35], leading to melanocyte deregulation and ultimately to neoplastic changes within the melanocyte [36,37]. Hence, cutaneous melanoma has a strong association with unprotected exposure to UV sunlight, and this is the most significant contributing factor to the disease process [38]. Concurrently, UV light exposure is also the most modifiable risk factor in melanoma development. The use of artificial light for cosmetic purposes exposes skin to increased amounts of UV-A compared to natural sunlight exposure, which would rationally explain the marked increase in cutaneous melanoma in recent years [39-41]. Furthermore, attitudes toward recreational sunbathing have evolved significantly over the past century, yet patient education regarding the value of applying sun protection factor (SPF) and the harmfulness of this activity lags behind. Previous blistering sunburn is renowned as a risk factor for melanoma, with a particular risk associated with intermittent sunburn during youth and childhood [38,42,43]. Severe sunburn experienced during childhood had been previously thought to play a greater role in melanoma tumorgenesis than similar affliction during adulthood [44]; however, a meta-analysis by Dennis et al. in 2010 noted severe sunburn during adulthood to be almost equally contributory to cutaneous melanoma diagnoses [45]. These environmental factors are well documented as the main contributors to malignant melanoma development; however, the concept of oncological progression is now considered a multifactorial process with a combination of genetic, epigenetic, and environmental factors, which are all known to be contributory [46]. Skin color or pigmentation is another undisputable non-modifiable risk factor influencing the likelihood of cutaneous malignant transformation [47]. Lighter skin pigmentation is associated with an increased risk of skin cancer, and subsequently, the Fitzpatrick skin spectrum was validated by dermatologists over the past four decades as a measure of sensitivity to sunlight and risk of cutaneous cancer development [48]. An increase in Fitzpatrick skin type is inversely associated with the risk of skin cancer [49]. Furthermore, chemical and thermal cutaneous burns have also been associated with increased incidence of melanoma transformation, although basal cell and squamous cell carcinomas are much more likely to be resultant [50]. Immunocompromised patients are considerably more likely to develop cancer than those with appropriate immunological responses to disease [51]. Consequentially, melanoma and other cutaneous skin cancers are more common in the immunocompromised population, and immunodeficiency is recognized as a significant risk factor for tumorgenesis. Within the tumor biology of cutaneous melanoma, suppression of the typical immunological response is paramount to malignant melanoma proliferation [52], which inevitably leads to oncogenesis within the melanocyte. Patients with xeroderma pigmentosum have the tendency to develop cutaneous and conjunctival melanomas [53]; this is due to a genetic defect in the ability to repair UV-light-induced DNA damage. Kraemer et al. noted that over 20% of patients with xeroderma pigmentosum subsequently progressed to develop cutaneous melanoma, highlighting the seriousness of this risk factor in potentiating tumor development [53].

As with other cancers, a synergistic interplay between various endogenous and exogenous factors precipitate oncogenesis with cutaneous melanoma [54,55]. The magnitude of the role of inheritance as a risk factor for cutaneous melanoma has recently been proposed in concordance with an enhanced understanding of molecular processes and oncogenesis, however remains considerably less significant than exogenous risk factors [56]. Various inherited patient factors are associated with melanoma development; the presence of naevi on the skin is greatly associated with malignant melanoma; having a large number of cutaneous naevi, having larger sized cutaneous naevi (>5 mm), and having “giant” naevi (>20 mm) are all associated with increased risk of melanoma development [57]. Between 29% and 49% of nonhereditary melanoma cases occur in the presence of a preexisting dysplastic naevus. Other described risk factors for melanoma proliferation include having light skin pigmentation (typically Fitzpatrick type-1 or type-2), fairer hair color, and green or blue iris pigmentation [57-62].

Repetitive sun exposure and UV-skin damage are highlighted as causative for cutaneous melanoma proliferation, while genetic inheritance is often overlooked as a causative risk factor. Despite the majority of genetic alterations associated with cutaneous melanoma tend to be “acquired,” the underlying presence of heritable mutation has a role in melanoma penetrance [63]. It is estimated that between 8% and 12% of patients with malignant melanoma have a family history of the disease [64-66]. Familial development of melanoma is inherited in a clear autosomal dominant inheritance pattern in “clusters,” with multiple family members being diagnosed in more than one generation [67]; of patients who have familial cutaneous melanoma, greater than 90% will have two family members who have had melanoma, whereas less than 10% will have three or more cases [68]. Inherited cases of malignant melanoma have been largely associated with mutation to a number of high penetrance genes, although mutation to the cyclin-dependent kinase inhibitor 2A (CDKN2A) gene located on chromosome 9 is documented as the most penetrant mutation to cause melanoma. In cases where patients have first-degree relatives with a previous melanoma diagnosis, 20%-40% will carry a CDKN2A mutation [69]. The CDKN2A gene is responsible for coding p16INK4A cyclin-dependent kinase inhibitor, and bi-allelic inactivation of this gene is strongly associated with invasive phenotype development [70,71]. Other high-penetrant genetic mutations associated with hereditary melanoma include cyclin-dependent kinase-4 (CDK4), telomerase reverse transcriptase (TERT), and the protection of telomere-1 (POT1), whereas the melanocortin-1 receptor and microphthalmia-associated transcription factors (MITF) are considered to have intermediate penetrance. Other inheritable mutations associated with developing malignant melanoma are BRCA1+associated protein-1/ubiquitin carboxy-terminal hydrolase (BAP1), adrenocortical dysplasia (ACD), telomeric repeat-binding factor 2-interacting protein (TERF2IP), and retinoblastoma (RB1) [69,72]. TERT promoter mutations have been demonstrated to induce proliferative changes in melanoma cells in conjunction with heterozygous CDKN2A alterations, and these have been described in in-situ disease [70].

Although we acknowledge both environmental and hereditary mutations are responsible for inducing cutaneous melanoma proliferation, the weight of somatic mutations is seen to harbor a larger effect in driving oncogenesis. Future direction for skin cancer research may elicit novel hereditary biomarkers responsible for driving the disease process, which may consequentially provide therapeutic avenues for patients to achieve an enhanced oncological outcome.

Tumor biology and pathophysiology

Cutaneous melanoma occurs when malignant transformation occurs in the pigment-producing melanocytes. Melanocytes are cells derived from the neural crest found typically in the basal epidermis, hair follicles, gastric mucosal surfaces, meninges, and uveal and choroidal layers of the eye [73]. As UV radiation causes damage to melanocytes, keratocytes in the skin produce melanocyte-stimulating hormone (MSH) that binds the melanocortin receptor 1 (MC1R) on the surface of the local melanocytes, which induces production and release of melanin. Secreted melanin acts locally as a protective layer, thus preventing further DNA damage via UV radiation [74]. We acknowledge the role of melanin as a protective shield in preventing alteration of genetic material within the threatened melanocyte.

A number of distinctive features of disease must be considered when attempting to classify and stratify cutaneous melanoma within Caucasian cohorts: the degree and duration of sun exposure prior to tumorgenesis, the anatomical locality of the tumor, the patient age at diagnosis, the types of oncogenic drivers, as well as the mutational load [75]. More elderly patients with background histories of chronic and high exposure rates to UV radiation (such as outdoor occupation or intense sunbathing in extremely sun-laden climates) are known to typically present with cancers of the head and neck region as well as diseases affecting the dorsal aspects of their upper limbs. These cancers are classically known to drive a high mutational load, secondary to the prolonged nature of UV sun damage, as this is the main environmental influence driving melanoma oncogenesis. The main genetic drivers of these chronic sun-damaged cancers are B-raf proto-oncogene (BRAF), neurofibromin-1 (NF-1), and NRAS mutations [75,76]. Alternatively, younger patients who have been exposed to limited or intermittent sun exposure to the trunk and proximal extremities are usually associated with melanoma-associated BRAFV600E mutation with lower mutational loads [76,77]. Furthermore, it has been noted that differing histopathological subtypes of cutaneous melanoma may evolve from varying precursor lesions in these younger patients and may involve various genetic mutations, making detection extremely challenging [36]. This inconsistent manner with which these cancers proliferate is further complicated by a number of other factors: whether initial tumor development occurs from precursor naevi; whether it follows a single, fluent transitionary stage; and whether this transformation may be marked by a variety of genetic mutations detected once a tumor undergoes histopathological and immunohistochemical evaluation. BRAF mutations have been noted to be present in as many as 80% of benign naevi in human cutaneous tissue, resulting in limited melanocyte proliferation through the oncogene mediated activation of cell senescence [78,79]. Studies have illustrated the benign nature of such naevi as they remain dormant for decades posing little oncological threat [80], indicating the BRAF mutation alone may be insufficient to potentiate melanoma tumorgenesis [36]. This further highlights the significance of the impact of exogenous triggers such as sun damage in triggering cutaneous melanoma proliferation. When mutation in this setting does occur, mutation to key genetic regulators in the oncogenic cascade such as CDK2NA or TERT is typically associated with oncogenesis. Reverting to the older, long-standing melanoma patients, these cancers seem to rarely occur from preexisting naevi. These are more commonly derived from de novo disease, or dysplastic lesions, and are subject to a considerably different panel of mutations [36]. In clinical practice, we rely hugely upon histopathological evaluation as the mainstay of cancer diagnosis, oncological management, and indicative of prognosis, providing stratification of malignancy to guide resection and treatment regimens. However, dependence upon histopathology alone leads to equivocal characterization and inappropriate risk stratification on account of differing pathophysiological triggers driving oncogenesis [81]. Perhaps application of our novel appreciation of the genetic, epigenetic, and biological factors driving melanoma carcinogenesis may lead to improvements in melanoma diagnosis, early recognition of patients with increased risk of progression, and overall enhanced oncological outcome.

Malignant melanoma is one of the most aggressive cancers in humans and is renowned for its potential to metastasize with extreme efficiency [7]. The leading cause of death in melanoma patients is distant metastasis, which is a consequence of the powerful metastatic potential of the disease [82]. Melanoma metastasis occur in a rapid, overwhelming nature, which is largely due to a combination of factors; genetic mutations make metastasis favorable; the cancers make alterations to the local biology of the microenvironment in which it resides and the overexpression of certain factors encouraging tumor invasion of surrounding tissue [83-85]. Proteases known as matrix metalloproteinases (MMPs) are overexpressed and cause degradation of the extracellular matrix and basement membrane within local cell structure, leading to the release of cytokines and matrikines producing favorable conditions in the local environment for tumor cell infiltration and hematogenous spread [84-87]. Alterations to the tumor and local microenvironment are mediated by genetic alterations and activation of the nuclear factor (NF)-kB pathway, a cascade leading to MMP-9 stimulation via the osteopontin (OPN) protein. MMP-9 overexpression is observed in cutaneous melanoma at times of metastasis and is initiated by an endogenous methylation phenomenon causing increased expression [88]. OPN is a key player in the tumor microenvironment within melanoma and has a fundamental role in disease development and progression [89]. OPN expression is first noted in staining on immunohistochemical evaluation of cutaneous melanoma tissue at the time of initial disease development and progression and is strongly correlated to metastasis in melanoma [90]. When making consideration for the natural history of cutaneous melanoma, these biomarkers play a fundamental role in initiating melanoma in the local tissue microenvironment and are pivotal in provoking disease metastasis.

Cutaneous melanoma carries one of the greatest burdens of somatic genetic alterations of all cancers [91]. Generally, the most commonly seen somatic genetic mutations in cutaneous melanoma are of course accountable to sun exposure, and these typically are those responsible for control and maintenance of central cellular activities such as growth and metabolism, replication, resisting apoptosis, cell cycle control, and proliferation [92]. Manipulation of these forms the foundation for targeted therapies applied to malignant melanoma in clinical practice. Somatic genetic mutations alluded to above typically diverge into disruption of one of these two pathways: the mitogen-activated protein kinase (MAPK) signaling pathway or the phosphoinositol-3-kinase (PI3K) cascade [93]. MAPK activation has been recognized as a key regulator of tumor biology in malignant melanoma and is the most common dysfunctional pathway within melanoma tumorgenesis, typically via the BRAF or NRAS mutations [94,95]. The MAPK pathway plays a crucial role in transporting extracellular signals, such as key regulatory proteins and hormones to the nucleus of cells, allowing for the expression of genes that are crucial to cell proliferation and differentiation [96]. Alternatively, PI3K signal transduction mostly involves cellular homeostasis and is recognized as the second most affected molecular pathway by somatic mutations in cutaneous melanoma [97].

The vast majority of patients diagnosed with cutaneous melanoma possess a dysfunctional MAPK pathway driving their disease. Melanoma proliferation typically relies on malfunction of signaling within the MAPK pathway based on the responsibility of MAPK in regulating the functional cell cycle while inhibiting apoptosis [96,98]. There are a considerable number of mechanisms responsible for MAPK signaling conduction malfunction; however, BRAF is by far the most common causative somatic mutation [99]. BRAF is a serine/threonine kinase, encoded on chromosome 7q34 responsible for activation of the MAPK signaling pathway in normal cell physiology [99,100]. The BRAF protein is composed of 766 amino acids organized into three major domains or regions, two for regulatory purposes and the third for catalytic phosphorylation [101]. Somatic BRAF mutation is seen in approximately 50% of melanomas, which are typically consequences of sun exposure damage [76]. In excess 80% of BRAF mutations detected in malignant melanoma are missense mutations, which cause the valine amino acid at position 600 to be substituted to glutamic acid (V600E); 5%-12% are valine to lysine (V600K) and less than 5% are valine to aspartic acid (V600D) or valine to arginine (V600R) [101,102]. Once V600E mutation occurs, there is a considerable structural deformity of the BRAF protein as the hydrophilic, polar glutamic acid replaces the hydrophobic valine amino acid. This results in an abnormal flip of the catalytic domain causing generation of a constitutively active conformation with kinase activity in excess of 500-fold higher than wild-type BRAF kinase [103,104]. The clinical significance of BRAF V600E detection is its sensitivity in predicting those who will respond positively to rapidly accelerated fibrosarcoma (RAF) inhibitor therapeutic strategies. However, these patients consistently develop disease progression after a variable time period, with a subset of patients developing primary resistance to these medications [105,106]. The majority of BRAF mutations not involving V600E act in a similar manner through dysfunction of the glycine-rich loop and activation of segment interaction, subsequently increasing BRAF kinase activity [104]. A coherent understanding of MAPK regulation at a molecular level is crucial, and acknowledgment of the various defects in the MAPK cascade is a significant driving force in melanoma oncogenesis.

There are a number of less penetrant mutations associated with melanoma proliferation: NRAS mutation is the next most common cause of aberrant signaling within the MAPK pathway and may be responsible for as many as 30% of somatic mutations affecting the MAPK cascade [107]. NRAS and BRAF mutations are considered to be somewhat mutually exclusive, with less than 1% of patients with cutaneous melanoma having co-mutations [108]. For the majority of cases, NRAS mutations are missense mutations of codons 12, 13, and 61, all of which influence GTPase activity; however, mutations to codon 61 alone account for up to 80% of NRAS mutations in cutaneous melanoma disease [104]. In cells with RAS mutation, the GTPase is locked in an active state providing continuous activation of NRAS signaling inducing downstream signaling within this pathway [109]. Increased signaling here leads to increased signaling through MAPK and PI3K pathways. NF-1 is a tumor suppressor gene (TSG) responsible for regulatory control of the RAS family by inactivating the RAS-guanine triphosphate complex, leading to downstream inhibition of RAS [110,111]. NF-1 has been described as the third most common mutated TSG seen within those who develop cutaneous melanoma, and once more, loss of NF-1 leads to increased signaling within the MAPK and PI3K pathways due to hyperactivation of the NRAS protein [110,112]. This form of mutation has been more commonly described in the pathogenesis of chronic sun-exposed skin and typically occurs in combination with other mutations such as NRAS or BRAF [113,114]. Approximately 2%-8% of malignant melanoma are seen to have somatic mutations within the receptor tyrosine kinase KIT gene, which subsequently leads to proliferation of disease via the PI3K and RAS pathways [115,116]. These mutations have a moderate association with acral melanoma and are associated with intermittent sun exposure.

## Conclusions

There are a number of key drivers in melanoma tumorgenesis, all of which have a variety of phenotypic characteristics. Although somatic mutational changes within the BRAF, NRAS, NF-1, and KIT genes are seen to deregulate tumor biology within the melanocytes, acknowledgment of the contributory role of inherited factors is also due. As modern science grapples with gaining an increased understanding and appreciation for the pathogenesis driving malignant melanoma, we recognize the modern advances in therapeutic strategies for treating the disease. Although the details underpinning the oncogenesis within melanoma are still not fully understood, the authors remain hopeful for the breakthrough identification of novel tissue biomarkers and therapeutic targets, which may enhance future patient outcomes within those diagnosed with cutaneous melanoma.
